# Motorpoint Heatmap of the Hamstring Muscles to Facilitate Neuromuscular Electrical Stimulation

**DOI:** 10.1007/s10439-024-03657-z

**Published:** 2024-11-28

**Authors:** J. Flodin, P. Amiri, R. Juthberg, P. W. Ackermann

**Affiliations:** 1 Integrative Orthopedic Laboratory, Section of Orthopedics and Sports Medicine, Department of Molecular Medicine and Surgery, Karolinska Institutet, Stockholm, Sweden; 2 Department of Trauma, Acute Surgery and Orthopaedics, Karolinska University Hospital, 171 76 Stockholm, Sweden

**Keywords:** Electrical stimulation, Motor point, Hamstring muscles, Motor unit, Muscle stimulation

## Abstract

The effectiveness of neuromuscular electrical stimulation (NMES) depends on electrode placement, with placement over the motor points (MPs) being the most effective. This study aimed to determine the MP-distribution and establish a heatmap indicating the probability of finding a MP in different areas of the hamstring (H) muscles. Additionally, inter-individual variations in the number of MPs were investigated. Thirty-one healthy participants (15 females, 16 males), aged 18–65 years, were included. The individual anatomy of the H-muscles, including the biceps femoris (BF), semitendinosus (ST), and semimembranosus (SM), was determined using ultrasound. MPs were located using a MP-search pen. The thigh anatomy was divided into 70 3x3cm areas, and the probability of finding a MP in each area was calculated using the Clopper–Pearson test to create a heatmap. Regression analysis was employed to determine if patient characteristics were associated with the number of MPs. The two best areas, found over BF and ST, exhibited a 39% probability of containing a MP and were significantly more likely to contain a MP compared to 85% of the remaining areas (*p* < 0.05). Two areas over SM had a 29% probability of containing a MP. BF exhibited a significantly higher number of MPs compared to SM and ST (*p* < 0.001). Male sex and higher physical activity were independent explanatory factors positively correlated with the number of MPs over BF in the multiple linear regression (*R*^2^ = 0.38, *p* = 0.001). The MP-heatmap of the H-muscles can effectively facilitate NMES application by highlighting areas with a higher probability of finding a MP. Large inter-individual variations in location and number of MPs were demonstrated.

## Introduction

Neuromuscular electrical stimulation (NMES) is a rehabilitation method commonly employed by physical therapists for patients recovering from orthopedic injuries or neurological disorders [1, 2], aimed at preventing muscle atrophy during immobilization, stimulating blood flow, and strengthening muscles [2, 3]. While NMES on the quadriceps muscle has been extensively studied [4–12], research on its application to the hamstring (H) muscles remains limited [4, 6, 13].

Strengthening of the H-muscles has been associated with improved mobility, balance, and reduced fall risk among older adults [14], suggesting that NMES activation of these muscles could yield positive outcomes for patients. However, optimizing treatment effectiveness requires maximizing compliance through the use of the most comfortable and energy-efficient electrode placements on the skin [4, 15–17].

Previous studies have demonstrated that placement of electrodes over muscle motor points (MP) significantly reduces discomfort and minimizes the required intensity during NMES [4, 16, 17]. MPs can be identified, with relatively high accuracy and speed by trained clinicians, using a “MP search pen” [18]. However, the procedure can be time consuming and challenging, especially for patients who wish to self-administer NMES, thus, mostly not used in clinical practice [2, 18]. A more practical approach is to create motor point maps indicating the most common MP locations. While such maps exist for quadriceps and calf muscles [4, 17, 19, 20], limited research has focused on the H-muscles [4]. In addition, a map that includes the probability of finding MPs over different areas of the H-muscle is lacking.

MP locations are individualized and can vary quite significantly between individuals [4, 17, 19, 20], influenced by factors such as body composition [9, 21], sex [9, 22], and age [23]. However, it is unknown whether the location and number of MPs vary based on participant characteristics.

Our hypothesis was that the likelihood of identifying a MP varies significantly across different areas of the skin covering the H-muscles, including the biceps femoris (BF), semitendinosus (ST), and semimembranosus (SM). We believe that this can be effectively visualized through an anatomical heatmap, facilitating MP localization during H-NMES. The primary objective of this study was to identify and map the MPs of the H-muscles to create a MP-map indicating the probability of finding a MP in different areas of the posterior thigh. A secondary aim was to investigate how participant characteristics may affect the number of MPs.

## Methods

### Ethical Approval

The study was performed in line with the principles of the Declaration of Helsinki. Ethical approval was obtained from the Regional Ethical Review Committees (Dnr: 2019–04020) and (Dnr: 2021–05076).

### Participants

A total of 31 healthy participants (16 male, 15 female) (Table 1) were included in this randomized explorative study performed at the Karolinska University Hospital during 2021. The inclusion criteria were age between 18 and 65 years, and no known acute or chronic disease. All participants signed an informed consent form and filled in a questionnaire detailing the participant characteristics including age, sex, height, body mass, smoking, and physical activity level [24]. Exclusion criteria were BMI > 30kg/m^2^ pregnancy, skin ulcer, pacemaker, intracardiac defibrillator, advanced heart disease, kidney failure, or neuromuscular/metabolic disease.

**Table 1:** Demographics and characteristics of the participants

Variable	Mean (Standard deviation) (*n* = 31)
Sex, Female, *n* (%)	15 (48.4)
Age, (Years)	31 (11.7)
Height, (cm)	176 (9.27)
Weight, (kg)	74.4 (14.7)
BMI, (kg/cm^2^)	24.0 (3.64)
Body fat^c^	22.5 (8.96)
Smoker, *n* (%)	0 (0)
Physical Activity level^a^	4.61 (1.23)
Length of thigh (cm)	46.8 (2.21)
Circumference of thigh (cm)^b^	57.4 (5.94)

^a^Frändin/Grimby activity scale [1–6]. ^b^Circumference of the thigh measured at 50% of the distance between the crease of the knee and the posterior mid-thigh line. ^c^Body fat according to the US Navy method. BMI = Body Mass Index

### Measurement of the Thigh

The length and circumference of each participant’s thigh were measured. The length of the posterior thigh was measured from the popliteal fossa to the gluteal sulcus, and the straight line connecting these two anatomical landmarks was defined as the “posterior mid-thigh line” (PMTL) [6]. The circumference of the thigh was measured at 50% of the length of the PMTL.

### Physical Activity Level

The physical activity level (PAS) was estimated using Frändin/Grimby activity scale, scored from 1 (no physical activity) to 6 (heavy physical exercise several times/week) [25].

### Body Fat US Navy Method

Each participant's body fat was estimated using the US navy method, with a formula including the participants’ height, circumference of the neck, waist, and hips (only women), which is considered a reliable method for estimating body fat [26].

### Neuromuscular Electrical Stimulation

For the MP-search, we used a constant-current NMES device, Chattanooga Physio (DJO Nordic, Malmoe, Sweden), together with a MP-search pen (DJO Nordic, Malmoe, Sweden). The parameters of the stimulation and set-up for the MP-search were the same as described in our previous study where the MPs of the quadriceps muscle were mapped [20]. The reference electrode was placed in the middle of the anterior thigh.

#### Intensity of NMES

The NMES level (1–999) expressed on the NMES device represents a non-linear relationship to the stimulation intensity ranging from 4 to 120 milliampere (mA). The NMES level needed to find each MP was translated into mA based on confidential information from DJO Global.

### Ultrasound Scan for Muscle Identification

To determine in which of the three different muscles of the hamstrings (BF, SM, and ST) each specific MPs belonged to, the individual anatomy of each participant´s posterior thigh was identified using the musculoskeletal program on an ultrasound machine (Philips CX50, Andover, MA, USA). Directly prior to the examination, participants were asked to lie down on a gurney in a prone position, and the ultrasound probe was covered by a layer of ultrasound gel (GIMA, Italy) to optimize ultrasound signal transfer. A marker pen was used to mark the individual anatomy of the three muscles on the skin for each participant, and it was decided that the overlapping area of SM and ST was marked as the ST-muscle since ST is located closer to the surface.

### Muscle Motor Point Search

A MP was defined in the same way as in our previous study [20], as a location on the skin that resulted in a muscle twitch at the lowest level of stimulation compared to the closest surrounding area [27], and the locations were determined by visual inspection and palpation of the muscle [4]. This study used the electrophysiological definition of a MP, e.g., the location on the skin covering the skeletal muscle where the least amount of electrical current is needed to cause a muscle contraction [27].

The MP-search was performed over the area of the posterior thigh based on the marked regions from the ultrasound scan. The entire area was covered with a layer of conductive gel (Conductor transmission gel, Chattanooga, DJO LLC, Dallas). Prior to the MP-search, participants were familiarized with the sensation of the MP-pen. The participants were informed that they could end the test at any moment without stating a reason. The MP-search was performed according to the instructions provided by DJO Global and in line with the procedure used in previous studies [4, 17, 19, 20]. The detailed procedure of the MP-search is described in a previously published paper [20]. Briefly, a search of the entire marked area was performed with the MP-pen (continuous stimulation, 3 Hz frequency and 400 µs pulse duration) for each intensity level, starting at the lowest level (NMES level 1), and during the search increased by one NMES level stepwise, and when a MP was identified the location was marked and the MPs were ranked based on what NMES level they were identified on. The MP identified on the lowest NMES level for each of the three muscles was considered the “best” MP for respective muscle.

A new MP was not allowed within a 5 cm radial distance from an already marked MP. This distance was chosen in order not to denote the same MP twice and based on a clinically relevant perspective, i.e., in order to allow placement of an 3 × 3 cm electrode over each MP without the electrodes overlapping or the edges touching each other as described in detail in our previous study [20].

The goal was to find at least two MPs on each of the three muscles on each participant, and this was thought to be possible based on previous studies [4, 17]. The MP-search ended due to any one of the following reasons: (1) the participant requested the MP-search to stop, (2) the whole area was covered by marked out MPs and their corresponding radial circles, such that there was no more free area to search for MPs, or (3) no more MPs were found during five consecutive NMES level increases of the MP-search. All MPs on all participants were found within a range of 5–25 mA after the test ending due to one of the above reasons.

**Fig. 1:**
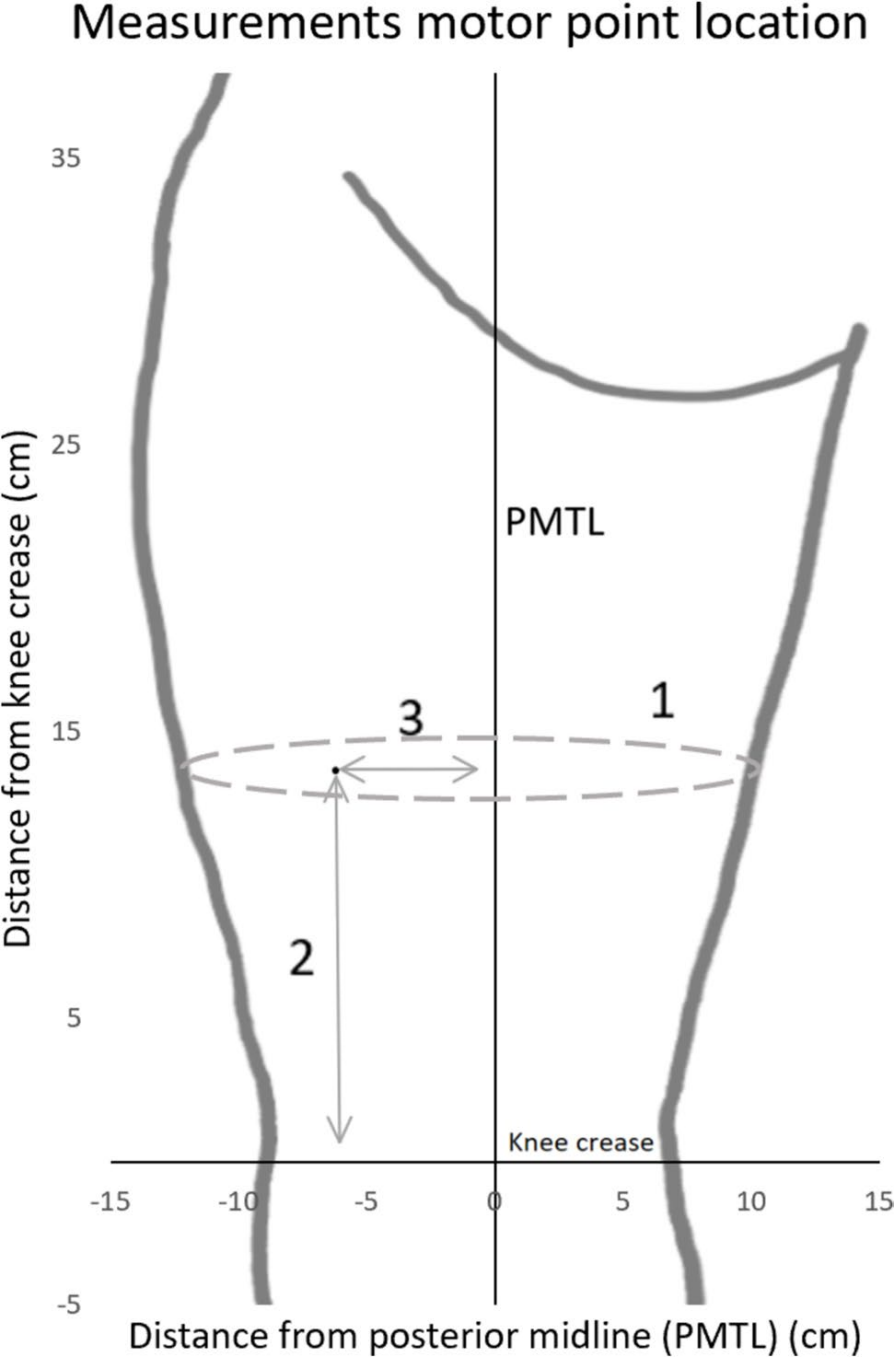
Assessment of motor point location. Description of how the location of each motor point was measured on the participants based on the posterior mid-thigh line (PMTL) and the knee crease. (1) The circumference of the thigh at the level of the point, (2) the longitudinal length from the knee crease, and (3) the horizontal distance from the PMTL

When the MP-search was finished, three measurements were performed for each MP to determine their positions: (1) the circumference of the thigh at the level of the point, (2) the longitudinal length from the knee crease, and (3) the horizontal distance from the PMTL (Fig. 1).

### Motor Point Maps

To create MP-maps representing the variations of leg sizes of all the participants, a “normalized thigh” was created based on the mean length and circumference of each participant’s thigh based on the same calculations used in our previous study [20] (Fig. 1). For each MP of each participant, the longitudinal percentual distance from the knee crease along PMTL was determined relative to each participant’s full PMTL length, and the horizontal percentual distance from PMTL was determined relative to the circumference where that MP was found. Thereafter, the relative locations of each of the participant’s MPs were added to the maps utilizing the normalized thigh based on the mean measurement of the 31 participants. Thus, all measurements and maps mentioned in the results and discussion are normalized.

**Fig. 2:**
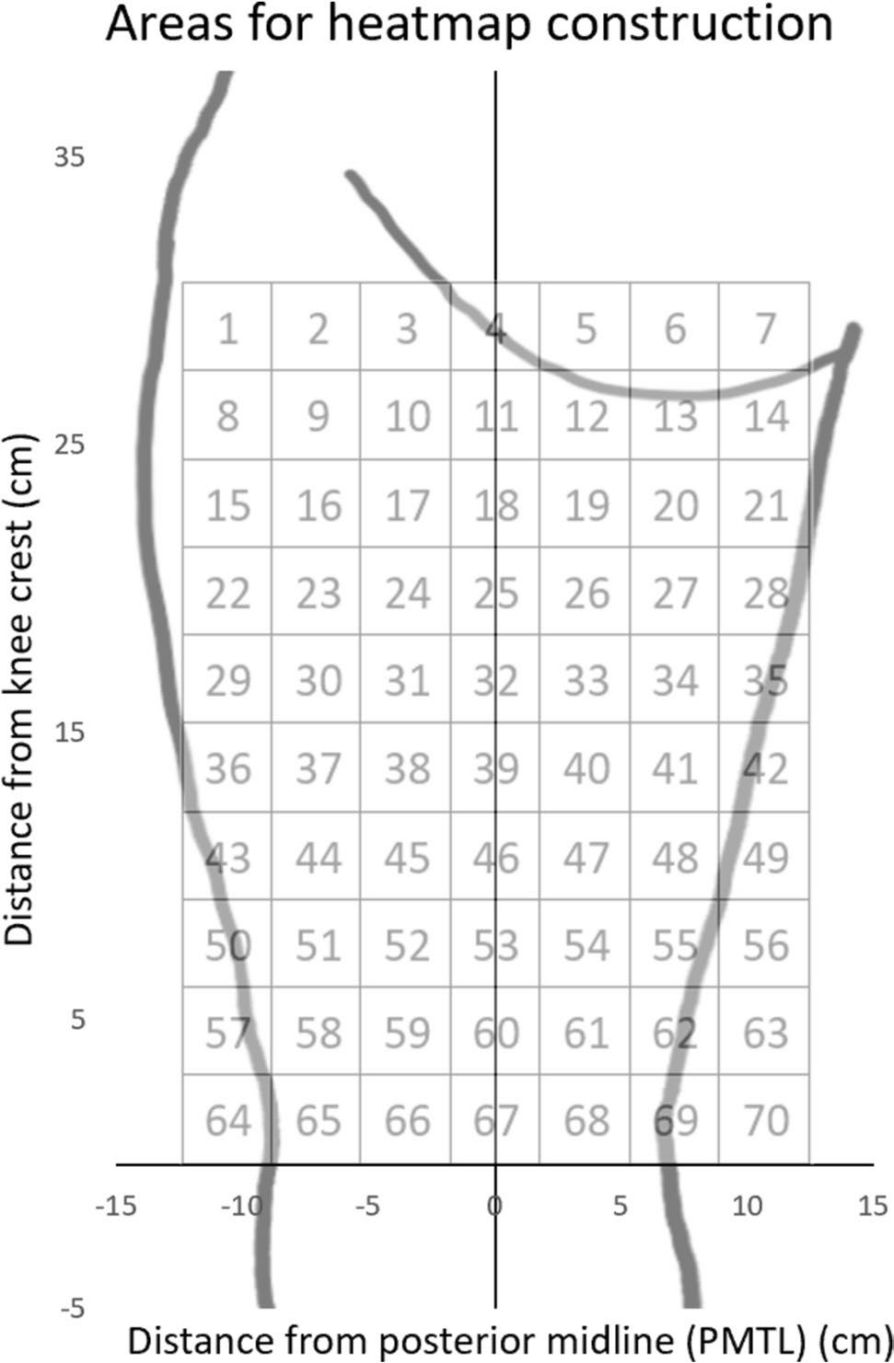
The areas in which the probability was calculated. The areas are sized 3x3 cm and are numbered from 1 to 70, starting from the proximal/lateral part of the posterior thigh. The areas outside the picture of the thigh correspond to lateral/medial regions of the thigh. PMTL = Posterior mid-thigh line

The “all MP-map” (including all MPs found on the whole H-muscle) and the “best MP-maps” (including only the best MPs from each of the three muscles) were created. In addition, to calculate the probability of finding a MP in different areas of the H-muscle, so-called “heatmaps” were created. Both an “all-MP heatmap” (heatmap including all MPs) as well as individual heatmaps for each of the three muscles (“BF-, ST- and SM-heatmap”) were created. For the heatmap construction, the area of the posterior thigh was divided into 70 3 × 3 cm areas (7 horizontal x 10 vertical) (Fig. 2). The 3x3 cm area size was chosen based on the same rationale as described in our previous studies [19, 20], e.g., the fact that an electrode size of 3x3 cm is the smallest electrode size used in clinical settings; furthermore, this electrode size eliminates any potential electrode overlap, given the 5 cm MP radius used.

### Statistical Analysis

Prior to the start of the experiment, the sample size was determined based on a power analysis with the significance level set at *p* < 0.05 and power at 80% regarding the primary outcome, i.e., a higher probability of finding a MP in one area compared to any other area in the heatmap. 29 participants were needed to obtain a significant difference, based on an estimated probability of finding a MP of 40% in one area compared to 20% in another area, and we set the final sample size to 31 participants.

The data were analyzed using SPSS version 27 (IBM Corp. Released 2020. IBM SPSS Statistics, Armonk, NY) in cooperation with a statistician. The distribution of the variables was checked using Shapiro Wilks test, and since normal distribution of the variables was found, descriptive data were reported with mean and standard deviation. In order to calculate the probability of finding a MP in each of the 70 areas, the areas containing one or more MPs were designated the value 1 and the areas without a MP the value 0 for each participant. For the all-MP heatmap, no difference was made between differently ranked MPs, meaning that all areas with at least one MP received a value of 1 independently of the rank. Thereafter, the Clopper–Pearson test was used to calculate the proportion of participants that had been designated the value 1 for any given area, i.e., the probability of finding a MP for any given area, presented with a 95% confidence interval (CI).

In addition to this, the paired* t* test was used to investigate the intensity of the “best-MP” compared to the mean intensity of other MPs that were identified, and the significance of correlations between the participant characteristics and the number of MPs found was determined by Pearson correlation. Thereafter, multiple linear regressions analyses (stepwise forward method, with an inclusion level of 0.05 and exclusion level of 0.10) were applied. This was done in order to investigate the unique relationships between the independent participant characteristics and the dependent variable number of MPs on the BF muscle. The variables considered for the regression analysis were sex, age, height, body mass, smoker, PAS, length of thigh, and circumference of thigh. All independent variables with significant correlation (*p* ≤ 0.05), or close to significant (*p* < 0.10), (e.g., sex, height, body fat, and PAS of the participants) were considered in the regression analysis. The significance level in all analyses was set at *p* ≤ 0.05 (two-tailed).

## Results

### Motor Point Map

**Fig. 3:**
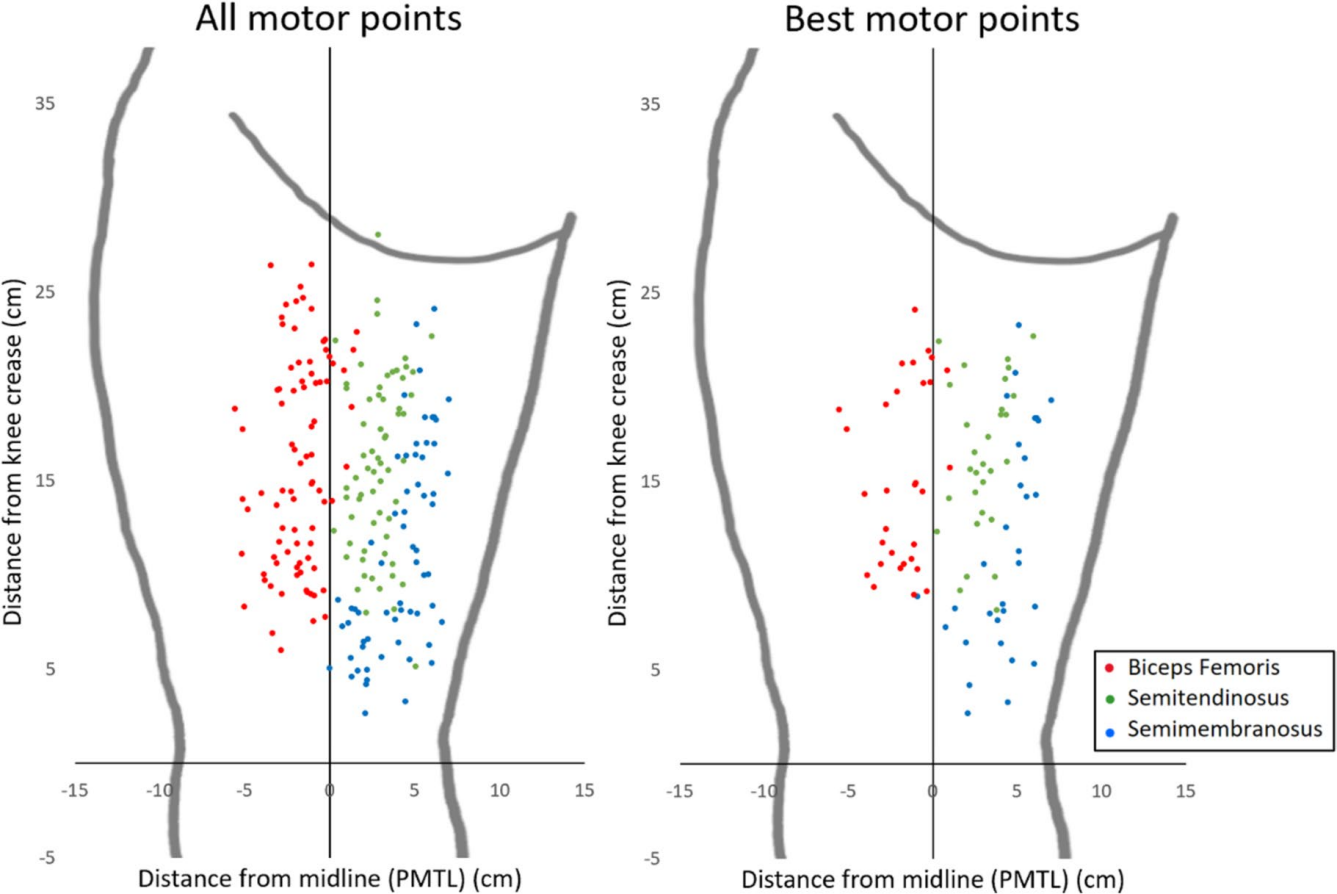
All- and best motor point (MP) maps. The maps display all MPs found (a) and the best MP found (b) on each of the three muscles. The different colors of the motor points indicate a location on one of the three muscles, biceps femoris (red), semitendinosus (green), and semimembranosus (blue). The horizontal axis represents the line corresponding to the knee crease, and the longitudinal axis represents the Posterior mid-thigh line (PMTL)

There was a significant inter-individual variation in the location and number of MPs observed over the H-muscles, generally ranging from 4 to 10 MPs, and specifically, one to four MPs over each of the three examined muscles (BF, ST, SM). The highest concentrations of MPs were observed in a central region of the posterior thigh along the BF long head and along the muscle border of the ST (Fig. 3a). The “best” MPs on each muscle, identified by the lowest possible intensity, are displayed in the “best MP-map” (Fig. 3b). For each of the three muscles, the “best” MP was found on a significantly lower level (*p* < 0.001) compared to all the other MPs on the respective muscle (Table 2).

**Table 2:** The current intensity at detection of motor points

Intensity (mA), mean (standard deviation)			
	Best MP	Other MPs	*p*-value
Biceps femoris	9.36 (2.81)	11.6 (2.62)	** < 0.001**
Semimembranosus	8.69 (2.38)	10.9 (2.18)	** < 0.001**
Semitendinosus	8.90 (2.71)	10.9 (2.46)	** < 0.001**

The “best” MP was defined as the MP discovered using the lowest NMES intensity, and “other” MPs as the mean value of all other MPs. All MPs from all 31 subjects were included. *P* values were calculated using paired *t* test.

*MP* motor point. *mA* milliampere.

### Motor Point Heatmap

**Fig. 4:**
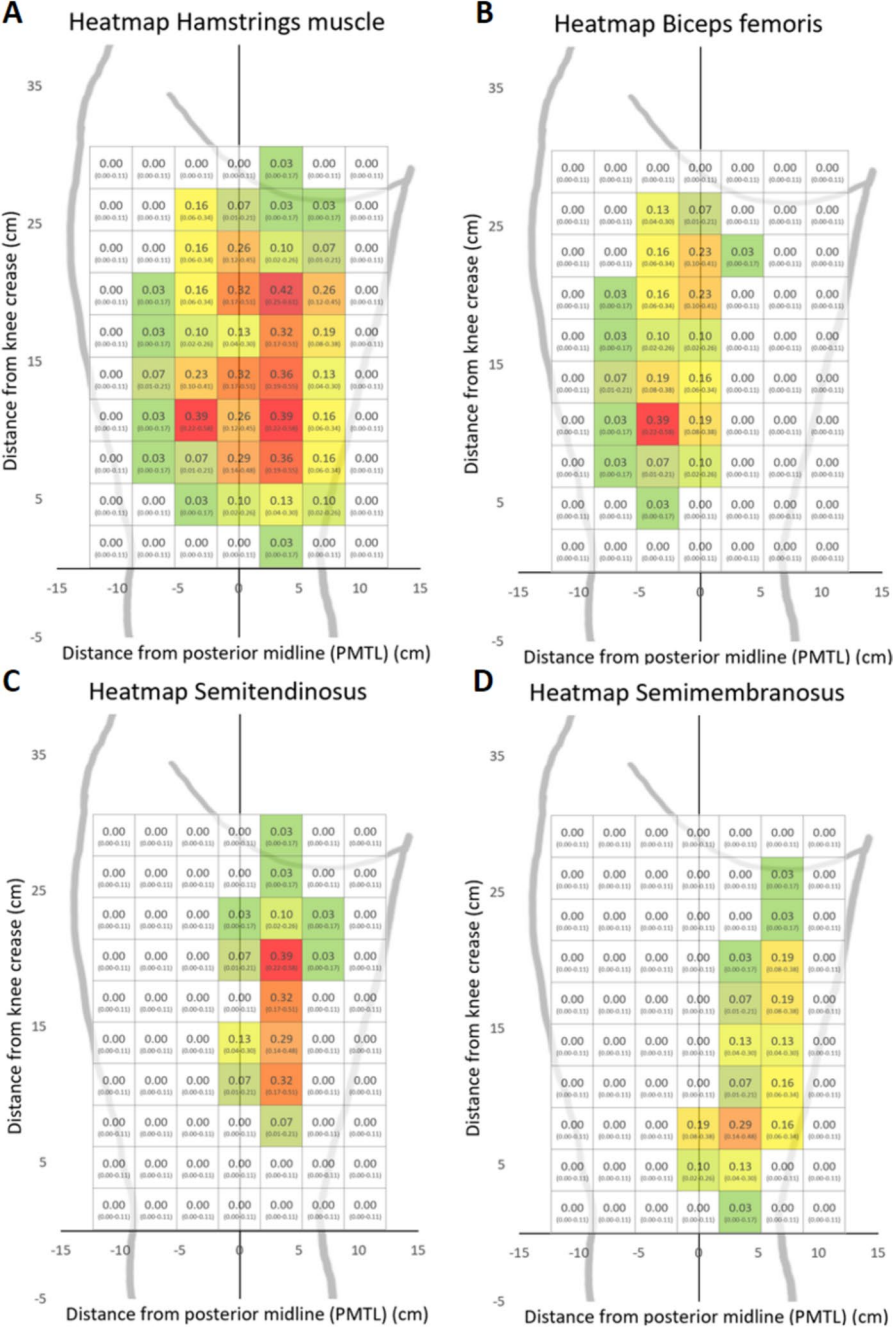
Heatmaps of MPs. The maps display the probability of finding any MP on the hamstring muscles (**a**), respectively, the probability of finding a MP on the biceps femoris (**b**), semitendinosus (**c**), and semimembranosus (**d**) in the different 3 × 3 cm areas, presented with 95% CI. The white areas indicate that no MP was found. The horizontal axis represents the line corresponding to the knee crease and the longitudinal axis represents the posterior mid-thigh line (PMTL). MP = motor point

Area 26, located in the central part of the posterior thigh (mainly containing ST, but also a small part of SM), demonstrated the highest probability (42%) of containing a MP over all H-muscles (“all-MP heatmap,” Fig. 4a). This area exhibited a significantly higher probability, with a 95% CI, of containing a MP compared to all areas with a mean probability lower than 25% (59 of the 70 3 × 3 cm areas) (Fig. 4a). In addition, seven other central areas of the posterior thigh showed a probability over 30% of containing a MP (areas 25, 33, 39, 40, 45, 47, and 54, Fig. 4a), which was significantly higher compared to all areas with a mean probability lower than 16% (51/70 areas). None of the white areas in Fig. 4 contained any MP.

All MPs found over the posterior thigh represent one of the three muscles investigated (SM, BF, ST), and an individual heatmap for each of the three muscles displayed the probability of finding a MP in the 3 × 3 cm areas for each muscle (Fig. 4b–d). The BF heatmap displayed 39% probability of finding a MP in area 45, which was a significantly higher probability of finding a MP than all areas with a probability lower than 22%, i.e., all areas except areas 18 and 25 (Fig. 4b). The ST heatmap exhibited four areas (26, 33, 40, and 47) with a probability between 29–39% of finding a MP, significantly higher compared to all other areas (calculated with 95% CI) (Fig. 4c). The SM-heatmap displayed 29% probability of finding a MP in area 54, which was, with 95% CI, significantly higher probability compared to all areas with a mean probability lower than 14% (Fig. 4d).

### Number of Motor Points

**Fig. 5:**
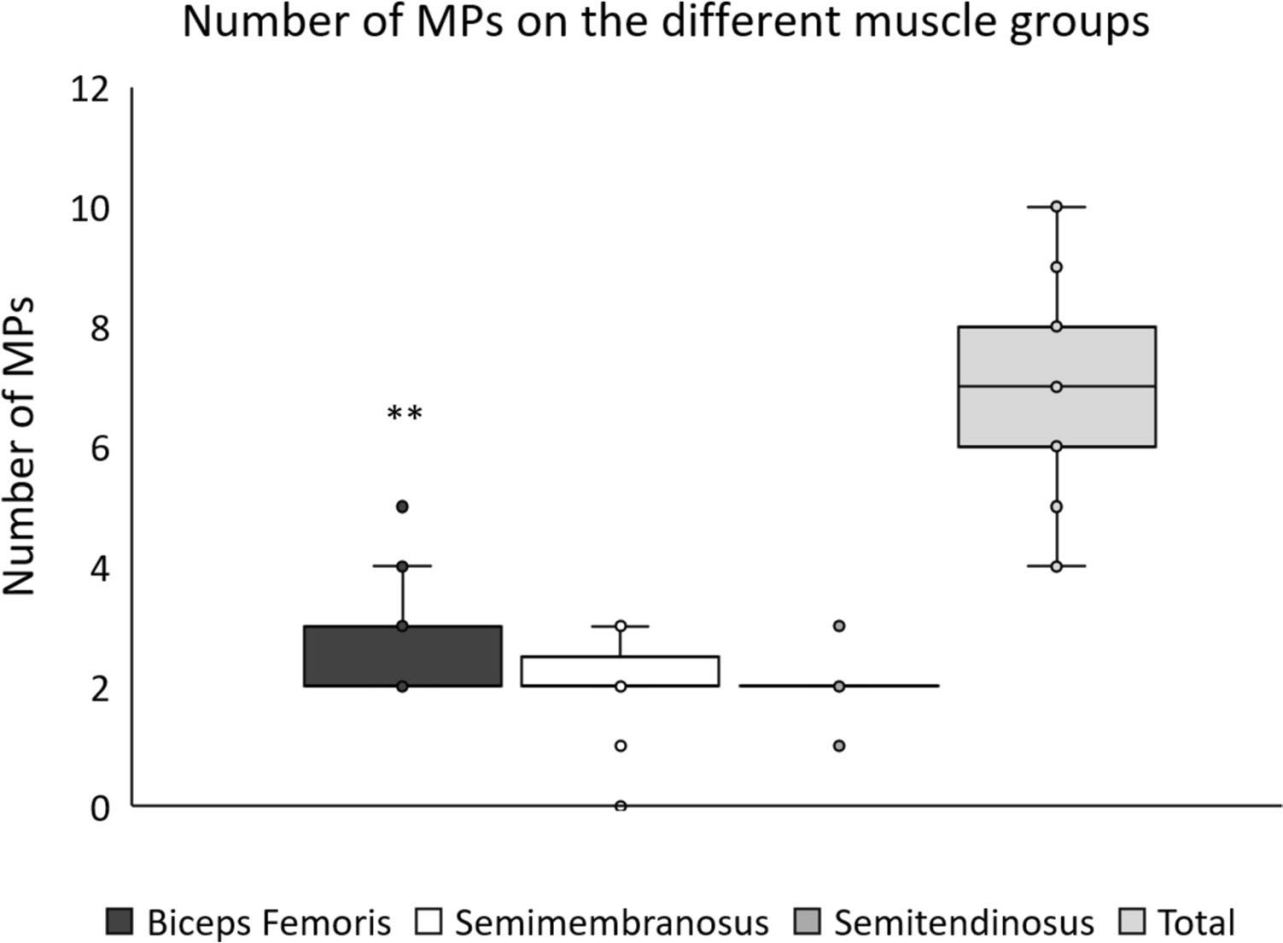
Boxplot of the number of MPs on the hamstring muscles. The number of MPs found on biceps femoris, semimembranosus, semitendinosus, and total number of MPs, respectively, on the 31 participants according to the description in the methods sections. **: *p* < 0.001 compared to the other two muscles. MP = motor point.

A mean total of 6.8 (±1.47) MPs was found over the whole H-muscle group. A significantly higher number of MPs was found over the BF, 2.68 (±0.83), compared to each of SM, 2.10 (±0.70), and ST, 2.03 (±0.41) (*p* < 0.001) (Fig. 5).

The number of MPs found over the BF muscle was significantly correlated to the sex, height, PAS, and body fat of the study participants (Table 3). These variables were entered into multiple linear regression analyses, which showed that male sex and higher PAS were independent factors, positively correlated with higher numbers of MPs of the BF muscle (*R*^2^ = 0.38, *p* = 0.001) (Table 3). For the SM and ST muscles, no significant correlations between number of MPs and participant characteristics were found.

**Table 3:** Correlation and multiple linear regression between the number of MPs found on the Biceps Femoris and participant characteristics

Number of MPs^a^	Pearson correlation	Multiple linear regression			
	(correlation coefficient, sig.)	Unstandardized coefficient		*T*	Sig.
		B	Standard error		
Constant		− 4.54	4.76	− 0.95	0.349
Sex, (male = 0, female = 1)	− 0.486, 0.006	− 0.687	0.247	− 2.79	**0.009**
Age, (years)	− 0.091, 0.625	–	–	–	–
Height, (cm)	0.467, 0.008	**	**	**	0.671
Weight, (kg)	0.220, 0.235	–	–	−	–
BMI, (kg/cm^2^)	− 0.047, 0.802	–	–	−	–
Circumference of thigh, (cm)^b^	0.043, 0.816	–	–	−	–
Body fat, (US Navy)	− 0.461, 0.009	**	**	**	0.473
Physical Activity Level^c^	0.460, 0.009	0.263	0.102	2.58	**0.015**
ANOVA		*r*^2^ = 0.383, *F*(2.30) = 8.68, Sig. = 0.001			

The participant characteristics that demonstrated a significant correlation to the number of MPs identified on BF were included in the multiple linear regression.

^a^Number of MPs over the BF according to the description in materials and methods. ^b^Circumference of the thigh measured at 50% of length of the PMTL. ^c^Frändin/Grimby activity scale [1–6]. *MP* motor point, *BMI* Body Mass Index, *BF PMTL* Posterior mid-thigh line. – variables not included in the regression model (sig. < 0.10 in Pearson correlation). **Variables not significant in the regression models.

## Discussion

In this study, MPs of the H-muscles were successfully localized and mapped, resulting in novel heatmaps that display the probability of finding MPs. These heatmaps serve as a valuable guide for electrode placement during H-NMES, particularly when a direct MP-search is impractical. In addition, the heatmaps enhance the efficiency and accuracy of the MP-search procedure, since, for example, physiotherapist using NMES in the clinic knows what area to focus the search on. The areas with the highest probability of finding MPs were approximately 7.5 cm (SM), 10 cm (BF), and 19 cm (ST) above the knee crease, and 2–5 cm medial (SM, ST), as well as 2–5 cm lateral (BF) of the PMTL.

The heatmaps of MPs on hamstrings, presented in this study, which include the probability of finding MPs in different areas of the posterior thigh, are innovative and could be used as a guidebook to speed up the procedure of locating MPs. A MP-search can be performed quickly and with relatively high accuracy by trained clinicians [18]; however, if performed by a layperson or health care staff without specific education in NMES, the MP-search can be more difficult to perform and also very time consuming [2]. In such cases, the use of heatmaps offers a practical solution, providing guidance on electrode placement when a formal MP-search is not feasible. By increasing the likelihood of correct electrode placement, these heatmaps have the potential to enhance treatment compliance and clinical outcomes of NMES treatment. Another main finding of this study was significant inter-individual variation in location and number of MPs over the H-muscles, with variations observed among different muscles (SM, ST, BF). The large inter-individual variation is consistent with previous studies on quadriceps [20], calf muscles [19], and H-muscles as studied by Botter et al. [4]. The large inter-individual variation of MP location emphasizes the critical step of identifying MPs, to induce effective and comfortable NMES. The high inter-individual variability in MP-distribution is also corroborated by earlier cadaveric studies on BF (short head) and SM muscles [4].

Notably, the distribution of MPs exhibited the highest concentration in the BF compared to the SM and ST muscles. The observed discrepancy in MP-distribution suggests that participant characteristics may influence the number of MPs to varying extent between different muscle groups. Previous studies have shown that factors such a muscle mass, subcutaneous fat thickness and BMI [7, 9, 21, 22, 28, 29] can affect NMES intensity level and comfort. Interestingly, males and more physically active individuals exhibited a higher number of MPs over the BF muscle, and together explained 38% of the variation in number of MPs over the BF. To the best of our knowledge, this study marks the first investigation into whether the number of MPs over the H-muscle is dependent on patient characteristics. The finding that the number of MPs on ST and SM were not correlated to physical characteristics presumably reflects the low number of MPs on these muscles creating a low variance for correlation.

However, a previous study on the quadriceps muscles [20] supports the notion that the variation in the number of MPs is dependent on the participants’ characteristics. That study found a significantly higher number of MPs in the quadriceps muscle for participants with a higher level of self-estimated physical activity, corroborating our observation in the BF muscle. Our finding that males had more MPs over the BF muscle diverges from the previous study on the quadriceps, which did not reveal any significant difference in MP number between males and females. This suggests that participant characteristics may affect the number of MPs differently across muscle groups. Other factors that may explain the observed discrepancies between muscle groups include the influence of NMES intensity level and comfort, which also are affected by participant characteristics such as muscle mass, subcutaneous fat thickness and BMI [7, 9, 21, 22, 28, 29]. It has been demonstrated that in patients with higher body fat, fewer MPs was found on the quadriceps muscle [20] and higher NMES intensity was needed for muscle contraction [9], likely because fat tissue impedes the conduction of the NMES current to the muscles [21]. In contrast to the previous study on the quadriceps muscle [20], body fat was not an explanatory factor to the number of MPs on the BF muscle. A possible explanation to this could be less variation in body fat percentage within the study group or also related to the fact that fat accumulation is more associated with the areas surrounding BF muscle rather than within the muscle themselves, however this warrants further investigation.

Another main finding of this study was that the distribution of MPs varied between the three individual H-muscles (SF, BF, SM). The various distributions and number of MPs could possibly be explained by anatomical variations in nerve supply and depth of muscle location. For instance, the long heads of the ST and BF are located more superficially than the SM [30]. In addition, the BF short head is innervated by the sciatic nerve common peroneal branch while the BF long head, ST, and SM are all innervated by the tibial nerve [17].

The areas shown in this study to contain the highest concentration of MPs over the H-muscles seem to conform with the findings of previous studies [4, 17]. Thus, area 26 in this study exhibiting the highest probability (42%) of finding a MP is also depicted in the study by Botter et al. [4]. Moreover, the observations of areas with the highest probability of finding MPs in each of BF, SM, and ST correspond to areas in the drawings of Botter et al. [4].

One potential limitation of this study was the reliance on visual examination to identify MPs, but the accuracy was enhanced by using ultrasound scans and palpatory assessment. Previous studies have demonstrated good reliability in assessing muscle twitch [6], also as additionally shown by visual determination of MPs (Gobbo et al., 2014, Botter et al., 2011), which is more accessible clinically than methods like electromyography. Another possible limitation was the exclusion of MPs found within 5 cm of each other during the search procedure. In addition, the NMES device used increased intensity in predetermined steps, potentially missing MPs between these levels. However, this likely had minimal impact on the results, as most participants had only one MP per muscle per intensity level. A notable strength of the study is the creation of a heatmap showing the probability of finding MPs in different regions of the H-muscles, offering valuable insights for electrode placement in clinical practice.

## Conclusions

This study demonstrated considerable inter-individual variation in MPs localization over the H-muscles, with higher MP numbers observed in more physically active individuals and males over the BF muscle. Despite individual differences, certain anatomic patterns of MPs distribution emerge, as revealed by the original heatmaps. These heatmaps provide valuable guidance for clinicians in electrode placement during NMES applications, enhancing treatment effectiveness and patient outcomes.

## Citation Diversity Statement

“Recent work in several fields of science has identified a bias in citation practices such that papers from women and other minority scholars are undercited relative to the number of papers in the field” (36). We have in this study recognized this and have worked to ensure that we have included references with fair gender and racial author inclusion.

## Abbreviations

BF: Biceps femoris
BMI: Body Mass Index
MP: Motor Point
NMES: Neuromuscular electrical stimulation
H: Hamstrings
PMTL: Posterior mid-thigh line
SM: Semimembranosus
ST: Semitendinosus
